# Supplementary material for: Anti PD-1/Anti PDL-1 Inhibitors in Advanced Gastroesophageal Cancers: A Systematic Review and Meta-Analysis of Phase 2/3 Randomized Controlled Trials
Source: Pharmaceutics. 2022 Sep 15;14(9):1953. doi: 10.3390/pharmaceutics14091953 (PMC9501109; doi:10.3390/pharmaceutics14091953)
Supplement: Supplementary file 1 [file pharmaceutics-14-01953-s001.zip › pharmaceutics-1833730-supplementary.pdf]

pharmaceutics

Supplementary Materials: Anti PD-1 /Anti PDL-1 Inhibitors in Advanced Gastroesophageal Cancers: A Systematic Review and Meta-Analysis of Phase 2/3 Randomized Controlled Trials

Kanak Parmar; Sai Subramanyam; Kristopher Attwood; Duke Appiah; Christos Fountzilas and Sarbajit Mukherjee

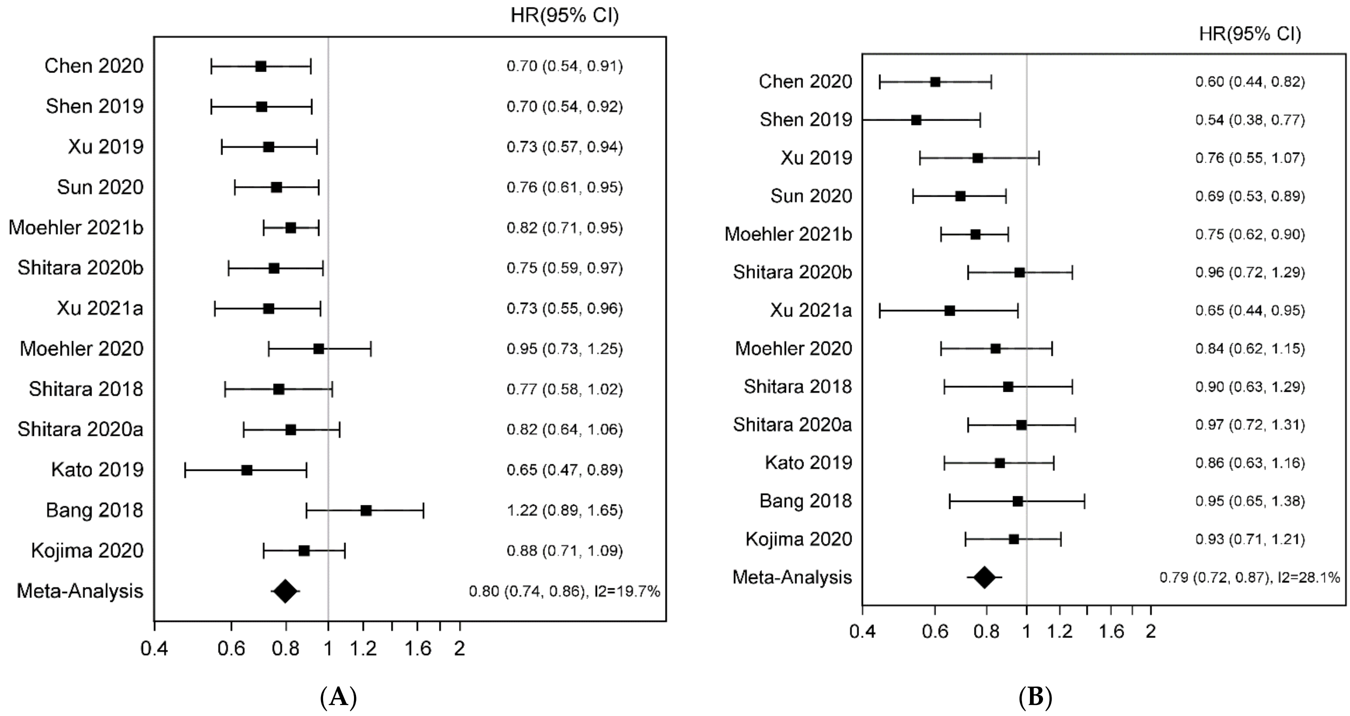

Figure S1. Overall population A: Forest plot for overall survival in the younger population; B: Forest plot for the overall survival for in the older population.

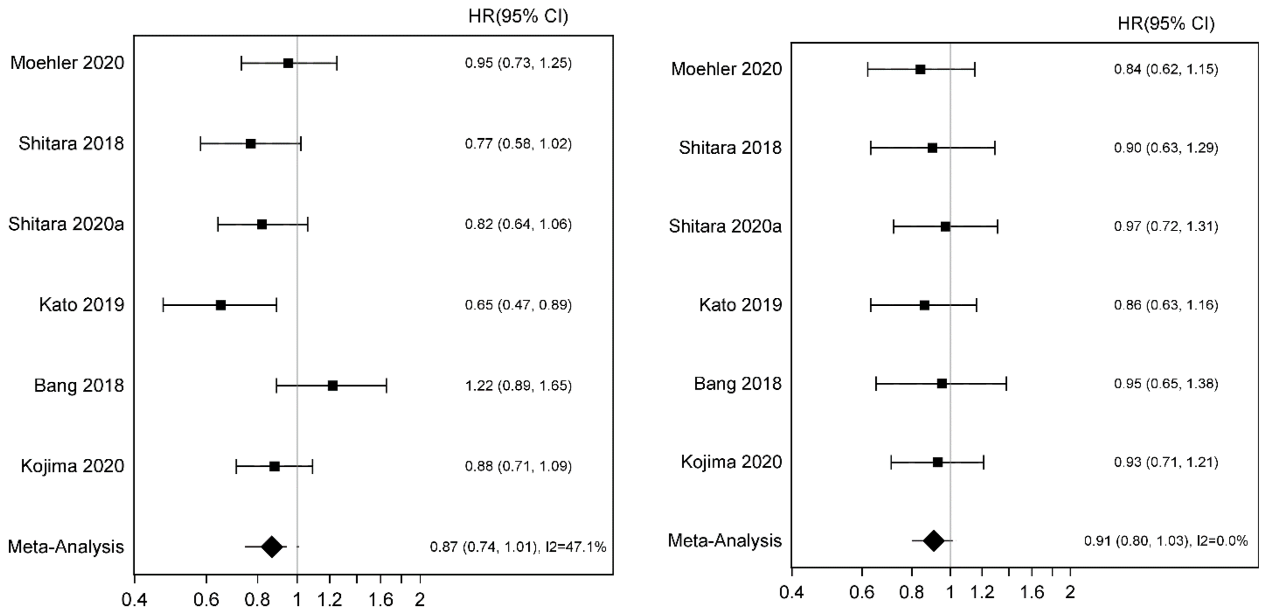

(**A**) (**B**)

Figure S2. Immune checkpoint inhibitor and chemotherapy versus chemotherapy alone A Forest plot for overall survival in younger age group B Forest plot for overall survival in the older age group

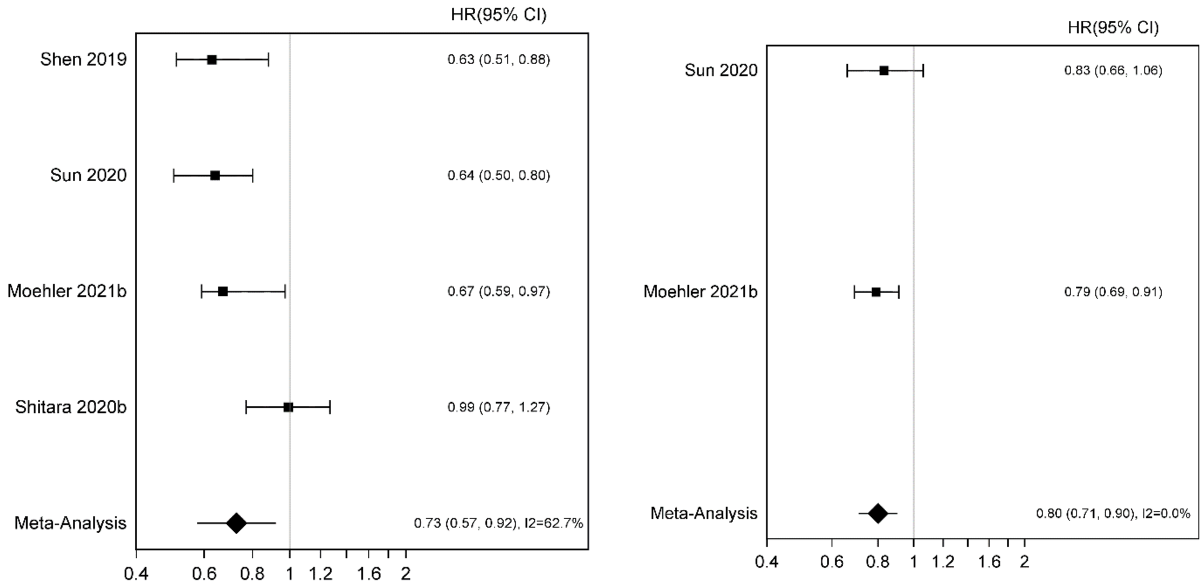

(**A**) (**B**)

Figure S3. Immune checkpoint inhibitor and chemotherapy versus chemotherapy alone; A. Forest plot for overall survival in Asians; B. Forest plot for overall survival in Whites

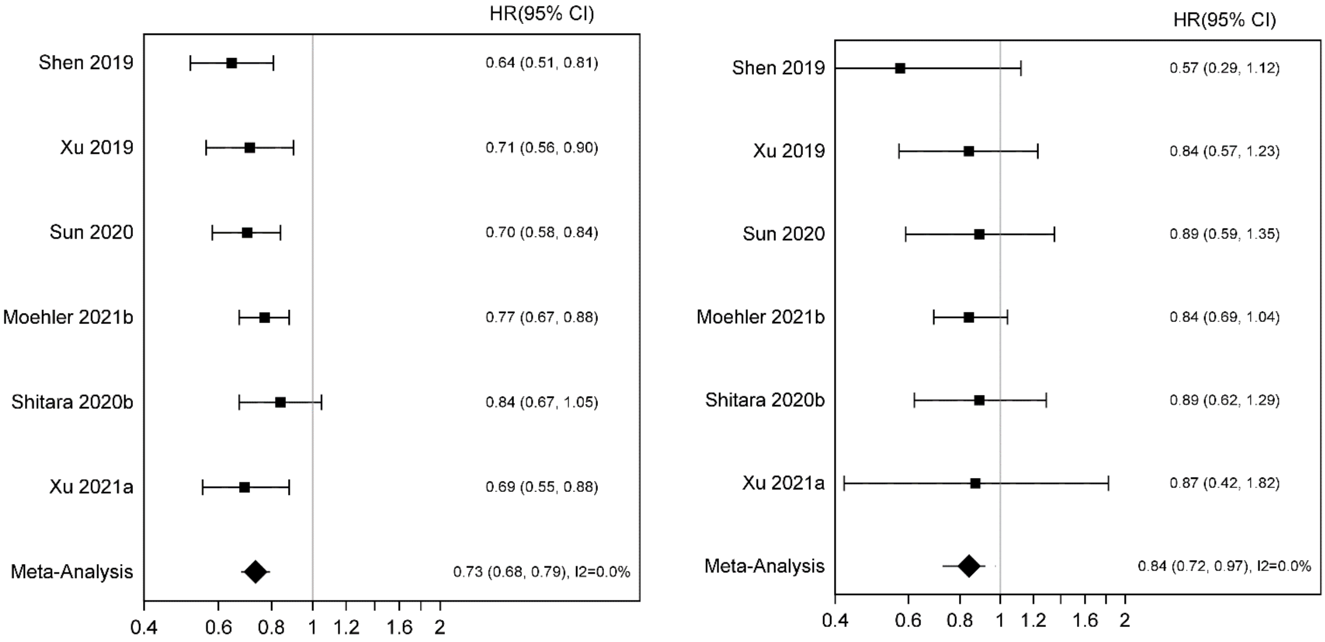

(**A**) (**B**)

Figure S4. Immune checkpoint inhibitor and chemotherapy versus chemotherapy alone; A. Forest plot for overall survival in males; B. Forest plot for overall survival in females

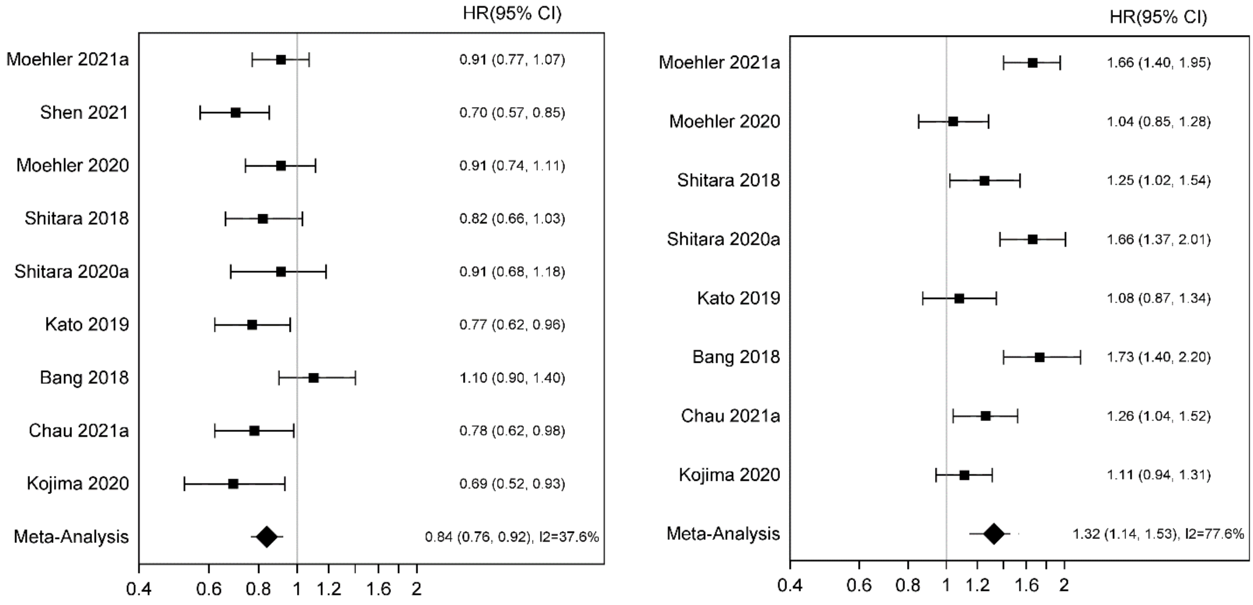

(**A**) (**B**)

Figure S5. Immune checkpoint inhibitor versus chemotherapy: A. Forest plot for overall survival; B. Forest plot for progression free survival.

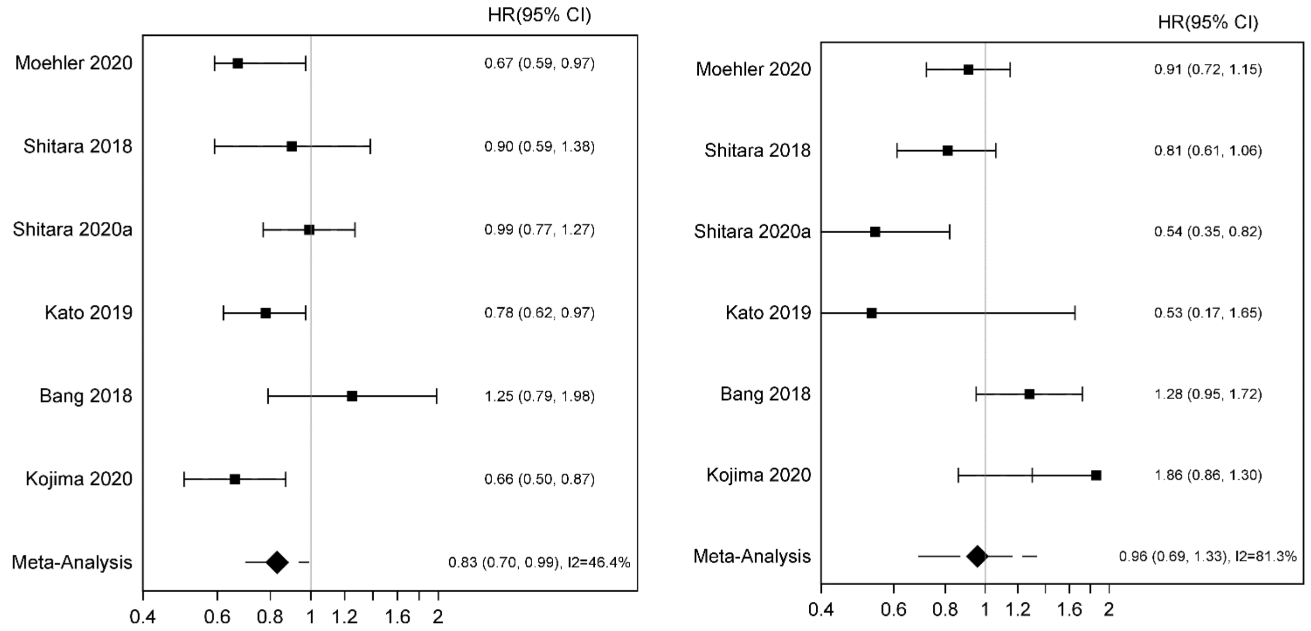

(**A**) (**B**)

Figure S6. Immune checkpoint inhibitor versus chemotherapy: A. Forest plot for overall survival in Asians; B. Forest plot for overall survival in Whites

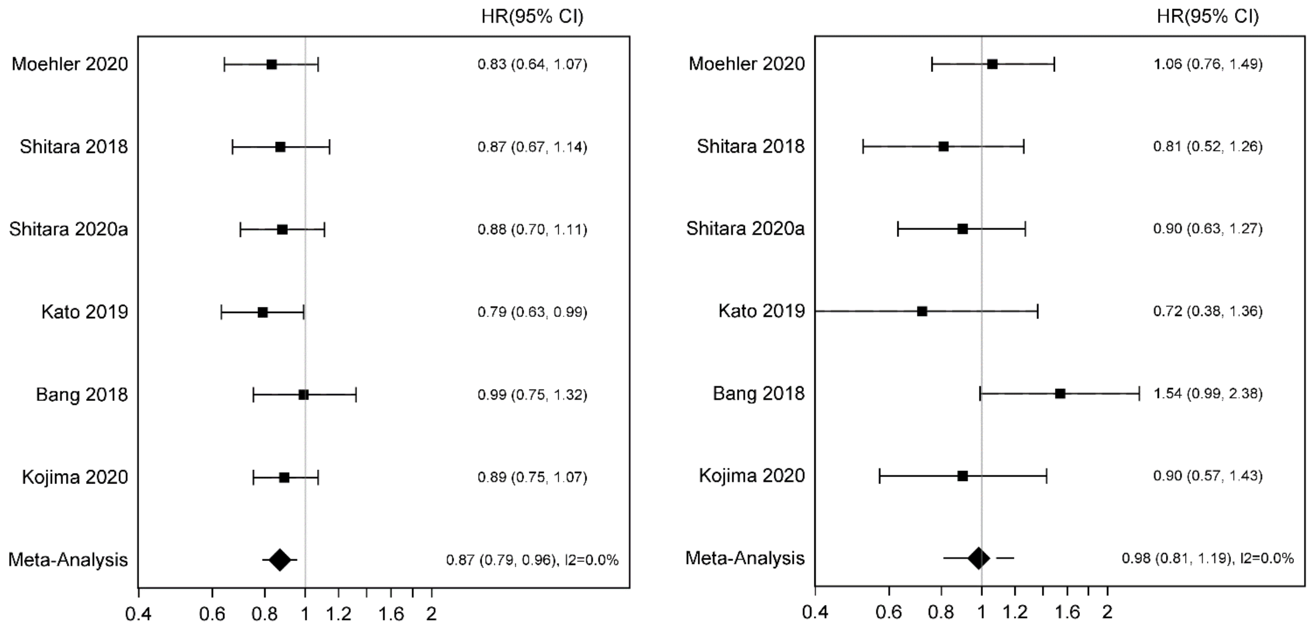

(**A**) (**B**)

Figure S7. Immune checkpoint inhibitor versus chemotherapy: A. Forest plot for overall survival in males; B. Forest plot for overall survival in females.

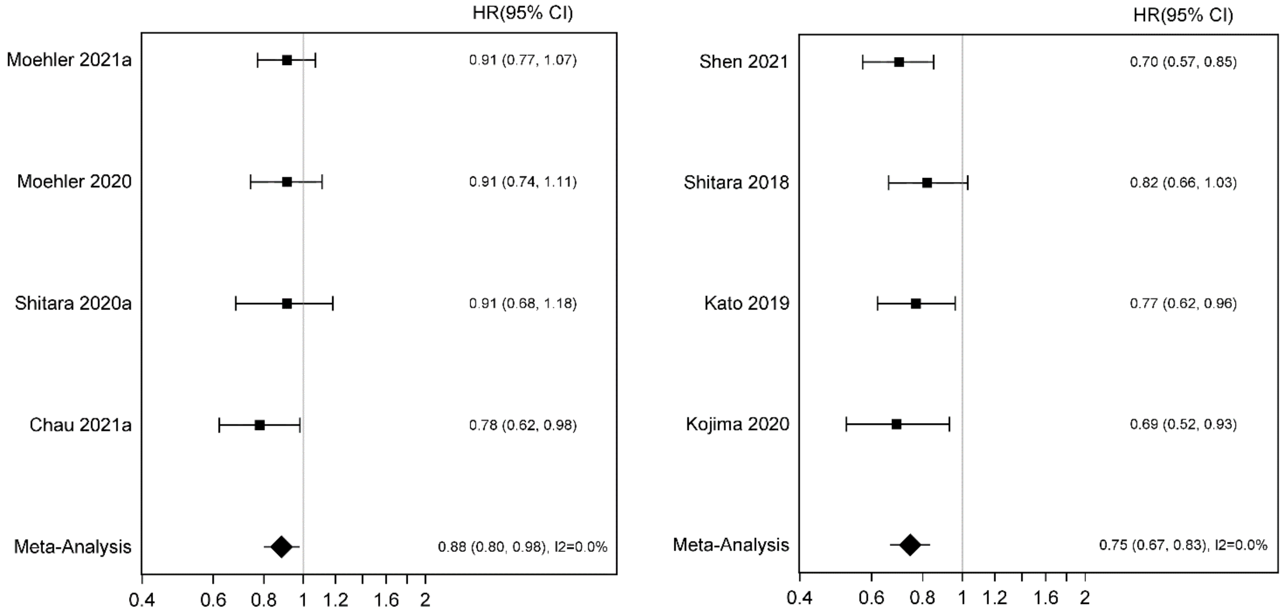

(**A**) (**B**)

Figure S8. Overall survival in immune checkpoint inhibitor versus chemotherapy: A. First line setting; B. Second line setting.

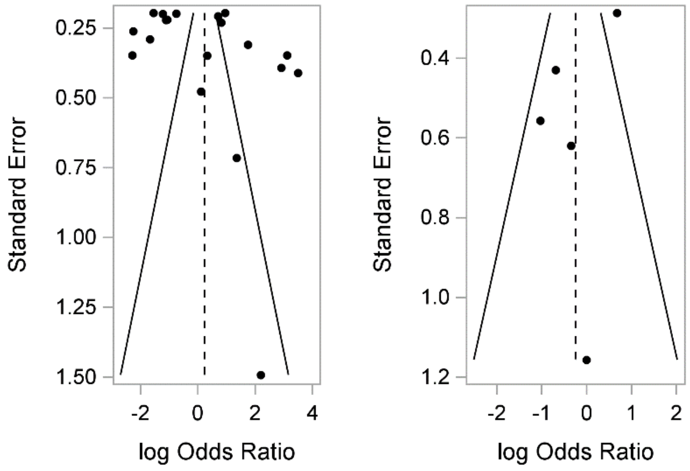

(**A**) (**B**)

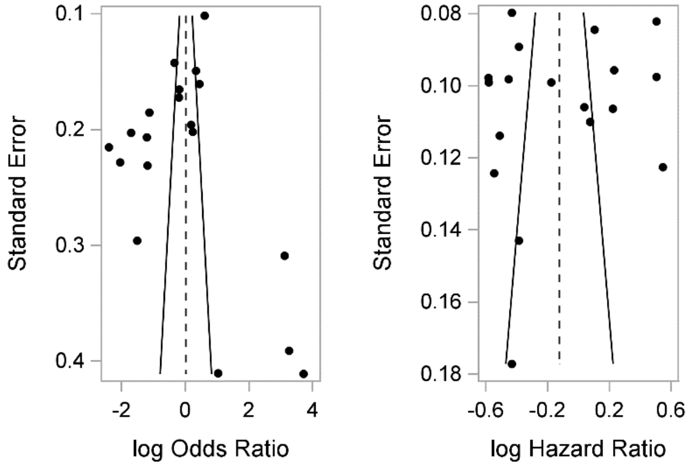

(**C**) (**D**)

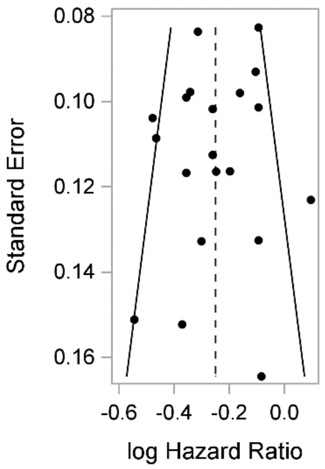

(**E**)

Figure S9. Funnel plots for A. Treatment related adverse event (TRAE); B. Any adverse event (AE); C. Grade 3 + related AE; D. Progression free survival, E. Overall survival
